# 16S Ribosomal Ribonucleic Acid Gene Polymerase Chain Reaction in the Diagnosis of Bloodstream Infections: A Systematic Review and Meta-Analysis

**DOI:** 10.1371/journal.pone.0127195

**Published:** 2015-05-21

**Authors:** Guoming Su, Zhuqing Fu, Liren Hu, Yueying Wang, Zuguo Zhao, Weiqing Yang

**Affiliations:** 1 Guangdong Provincial Key Laboratory of Medical Molecular Diagnostics, Guangdong Medical College, Dongguan, China; 2 Department of Microbiology and Immunology, Guangdong Medical College, Zhanjiang, China; 3 Department of Epidemiology and Health Statistics, School of Public Health, Guangdong Medical College, Zhanjiang, China; University of Leicester, UNITED KINGDOM

## Abstract

**Objective:**

We aim to evaluate the accuracy of the 16S ribosomal ribonucleic acid (rRNA) gene polymerase chain reaction (PCR) test in the diagnosis of bloodstream infections through a systematic review and meta-analysis.

**Methods:**

A computerized literature search was conducted to identify studies that assessed the diagnostic value of 16S rRNA gene PCR test for bloodstream infections. Study quality was assessed using the revised Quality Assessment of Diagnostic Accuracy Studies (QUADAS-2) tool. We calculated the sensitivity, specificity, positive likelihood ratio (PLR), negative likelihood ratio (NLR), diagnostic odds ratio (DOR) and their 95% confidence intervals (95% CI) for each study. Summary receiver operating characteristic (SROC) curve was used to summarize overall test performance. Statistical analysis was performed in Meta-DiSc 1.4 and Stata/SE 12.0 software.

**Results:**

Twenty-eight studies were included in our meta-analysis. Using random-effect model analysis, the pooled sensitivity, specificity, PLR, NLR, and DOR were 0.87 (95% CI, 0.85–0.89), 0.94 (95% CI, 0.93–0.95), 12.65 (95% CI, 8.04–19.90), 0.14 (95% CI, 0.08–0.24), and 116.76 (95% CI, 52.02–262.05), respectively. The SROC curve indicated that the area under the curve (AUC) was 0.9690 and the maximum joint sensitivity and specificity (Q*) was 0.9183. In addition, heterogeneity was statistically significant but was not caused by the threshold effect.

**Conclusion:**

Existing data suggest that 16S rRNA gene PCR test is a practical tool for the rapid screening of sepsis. Further prospective studies are needed to assess the diagnostic value of PCR amplification and DNA microarray hybridization of 16S rRNA gene in the future.

## Introduction

Bloodstream infections (BSIs) remain a major cause of morbidity and mortality especially in the Intensive Care Unit [1–5]. Moreover, inadequate antibiotic therapy is associated with higher mortality rates. Early microbiological diagnosis is of paramount importance for appropriate antibiotic treatment which increases the survival rate of patients [4]. Therefore, it is evident that a rapid, sensitive and specific diagnosis of BSIs is urgently needed.

Conventional identification methods have several limitations such as lack of rapidity and sensitivity. Blood cultures followed by conventional identification methods are currently the reference method for the detection of pathogens in blood. This well-established method can detect a wide range of microorganisms. However, disadvantages do exist, as the time to detection is often too long. After the detection of bacteria by conventional blood culture, identification and assessment of antibiotic sensitivity take at least a further 24 h [6, 7]. The sensitivity is also unacceptably low in the detection of pathogenic bacteria in cases of low-grade bacteremia, in cases where blood cultures are inoculated without adequate sample volume, and in cases where antibiotics are used before blood samples are taken [8, 9]. If blood cultures were negative, repetition of sampling would been required, while positive cases need further identification of the isolated microorganism using different culture media and biochemical tests [9]. For these reasons, development of detection methods that provide more rapid results and higher sensitivity is expected to optimize use of antibiotics.

An ideal diagnostic tool for BSIs should be rapid, sensitive and unaffected by antibiotic therapy. In recent years, polymerase chain reaction (PCR) assay using the 16S ribosomal ribonucleic acid (rRNA) gene has been used as a diagnostic tool in many setting [1, 9, 10]. This test is based on the rationale that 16S rRNA gene of bacteria comprises both conserved and variable regions [11]—the conserved regions are targeted by universal primers for identification of bacterial infection and the variable regions by genus or species-specific assays [2]. Amplified target regions may then be subjected to downstream applications such as sequence analysis and microarray hybridization [5, 9, 11, 12]. In addition, the PCR test has the advantages of amplifying minute amounts of DNA, even from nonviable bacteria [13], and costing less money than blood cultures in both negative and positive cases [9].

However, the results of these studies were variable although inspiring. Some studies revealed the diagnosis of BSIs by 16S rRNA gene PCR test with no less than 95% sensitivity and 95% specificity [1, 14, 15], whereas others reported low sensitivity values ranging from 41% to 90% [9, 16–18] and low specificity values ranging from 32% to 90% [2, 3, 19, 20]. Therefore, we performed a systematic review and meta-analysis to evaluate the accuracy of 16S rRNA gene PCR test compared with conventional blood culture in the diagnosis of BSIs.

## Materials and Methods

### Search strategy

This systematic review was performed according to the PRISMA Statement [21] (S1 PRISMA Checklist) and Cochrane Collaboration guidelines (http://handbook.cochrane.org/). A systematic literature search was performed for studies that assessed the diagnostic value of 16S rRNA gene PCR test for BSIs. We searched PubMed, Embase, the Cochrane Library, ClinicalTrials.gov (www.clinicaltrials.gov) and the World Health Organization International Trials Registry Platform search portal (http://apps.who.int/trialsearch/Default.aspx) up to March, 2015. The search terms were “RNA, Ribosomal, 16S”, “16S ribosomal ribonucleic acid gene”, “16S rRNA gene”, “16S rDNA”, “sepsis”, “bloodstream infections”, “bacteremia”, and “septicemia”. We gave the detailed search strategies in S1 Table. Our searches were not limited by publication date, country or language. The databases search was conducted independently by two authors (Guoming Su and Yueying Wang). To ensure comprehensive acquisition of literature, we also manually searched for any additional studies in the reference lists of retrieved studies and recent reviews.

### Inclusion and exclusion criteria

Studies were then included if they met the following inclusion criteria: (1) studies assessed diagnostic value of 16S rRNA gene PCR test for BSIs such as neonatal sepsis and bacteremia; (2) Blood cultures followed by conventional identification methods were used as the reference standard; (3)each study contained no less than ten specimens; and (4) Studies provided sufficient data to allow construction of two-by-two tables. If the same experimental results were repeatedly or multiply published, only the most informative publication was included. Relevant articles were excluded if they were review articles, meta-analysis, commentaries, letters, or case reports. Two reviewers (Guoming Su and Zhuqing Fu) independently screened studies according to eligibility criteria. Disagreements were resolved by consensus.

### Data extraction and quality assessment

Two reviewers (Guoming Su and Zhuqing Fu) independently extracted information from eligible studies using a predefined data extraction form, and then another reviewer (Yueying Wang) verified them. Disagreements between reviewers were resolved through discussion. The following information was subtracted from the studies: the first author, publication year, country, disease type, participant characteristics, specimen type, test methods, cut-off of index test, and data for a two-by-two table (true positive, false positive, false negative, and true negative information), respectively.

The quality of each study was assessed using the revised Quality Assessment of Diagnostic Accuracy Studies (QUADAS-2) tool (http://www.bris.ac.uk/quadas/). This tool comprises 4 domains that discuss patient selection, index test, reference standard, and flow and timing [22]. Each domain is assessed in terms of risk of bias by using signaling questions which are answered with“yes,” “no,” or “unclear”. And risk of bias is judged as “low”, “high”, or “unclear”. In addition, the first 3 domains are simultaneously assessed in terms of concerns regarding applicability which are also rated as ‘‘low”, ‘‘high”, or ‘‘unclear” with the similar criteria.

### Definitions of amplification methods

Amplification methods are defined as following: (1)PCR is defined as a conventional PCR amplification strategy that targets 16S rRNA gene in microorganisms. (2)Real-time PCR is defined that is an amplification method with a real-time monitoring system. (3)PCR-hybridization is defined as an amplification method followed by reverse hybridization or DNA microarray hybridization. (4)PCR-sequencing is defined that is an amplification method followed by sequence analysis. (5)RT-qPCR is a reverse transcription-quantitative PCR method for the determination of copy number of PCR templates such as RNA or cDNA in a PCR reaction. (6)FQ-PCR is a fluorescent quantitative PCR method for the determination of copy number of amplification cycles.

### Data analysis

All analyses were undertaken using the Meta-DiSc 1.4 [23] and Stata/SE 12.0 software [24]. The Spearman model was applied to assess heterogeneity caused by different cut-off threshold effects, while the heterogeneity that was caused by other factors was checked using Cochran-Q value and I^2^ test [25, 26] for diagnostic odds ratio (DOR). If heterogeneity (ρ <0.05 or I^2^ >50%) was statistically significant among studies, the random-effect model [27] was performed for the meta-analysis; otherwise, the fixed-effect model [28] was chosen. For each study, the following indexes of test accuracy were calculated: sensitivity, specificity, positive likelihood ratio (PLR), negative likelihood ratio (NLR), diagnostic odds ratio (DOR), and their 95% confidence intervals (95% CI). Summary receiver operating characteristic (SROC) curve was used to summarize overall test performance [29]. The area under the curve (AUC) and Q point value (Q*) were also counted to assess the overall performance of the diagnostic test accuracy [30]. In addition, subgroup and meta-regression analysis [31, 32] were conducted to explore the possible sources of heterogeneity among studies. Publication bias was inspected using Deeks' funnel plot asymmetry test [33]. A two-sided *p*-value of <0.05 was considered to be statistically significant.

## Results

### Search results

**Fig 1:**
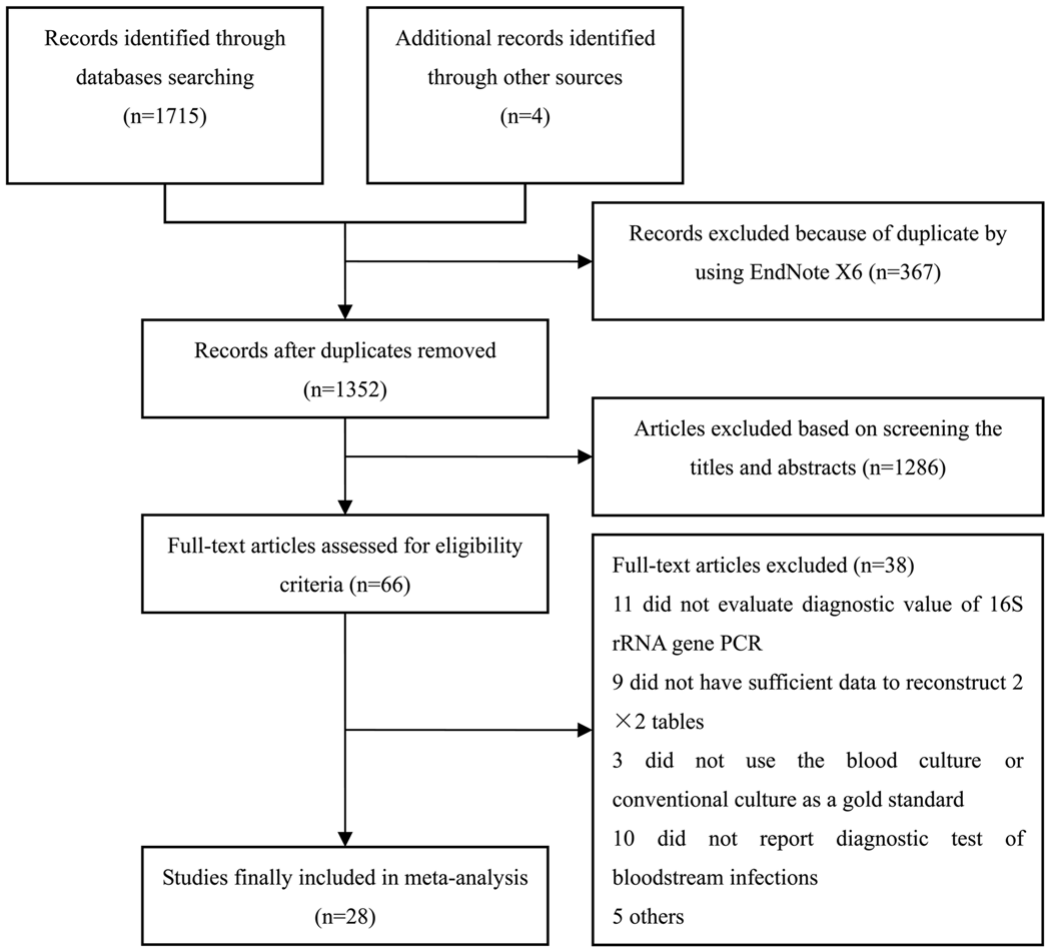
Flow chart of selection process for eligible studies.

A total of 1,715 titles and abstracts were found from initial searches of the electronic database. Four records were identified through reviewing the references of the other meta-analysis and reviews. Firstly, 367 records were excluded using EndNote X6 due to duplication. We applied the inclusion and exclusion criteria to filter out 1,286 records, because they were considered as review articles, meta-analysis, commentaries, letters, case reports, or records about apparently irrelevant to study question. Leaving 66 articles were eligible for further full-text review. Subsequently, additional 38 articles were further excluded after a full-text review. The list of full-text excluded articles, along with detailed reasons for exclusion, is presented in the supporting information (S2 Table). Finally, a total of 28 studies that met inclusion criteria were included in the present meta-analysis [1–5, 9, 10, 12, 14–20, 34–46]. The details of study selection flow are summarized in Fig 1.

### Characteristics and quality of the studies

The main characteristics of the included studies are shown in Table 1. Our meta-analysis included 28 studies which were published between 1997 and 2014. A total of 7,378 specimens were taken from infants to adults. Of 7,378 specimens, 219 were venous access ports [10], 10 were cerebrospinal fluids [38, 40, 41], and the rest were blood samples. All specimens were confirmed by conventional identification methods which were currently the reference standard in the diagnosis of bacterial infections.

**Table 1:** Characteristics of the studies included in the meta-analysis.

Study	Country	Disease type	Participant characteristics	Specimen type	Test methods	Cut-off	TP	FP	FN	TN
Liu CL, 2014[1]	China	Sepsis	Neonates	Blood	PCR	630 and 216 bp	95	28	0	583
Hassan RM, 2014[9]	Egypt	Bloodstream infections	All age groups	Blood	PCR-sequencing	Sequence similarity≥97%	58	21	11	198
Guembe M, 2013[10]	Spain	Bloodstream infections	Adult patients	Venous access ports	PCR-sequencing	Sequence similarity≥99%	12	53	3	151
Shaat SS, 2013[2]	Egypt	Sepsis	Neonates	Blood	PCR	1100 bp	17	7	0	26
Negoro E, 2013[12]	Japan	Bacteremia	NR	Blood	PCR-hybridization	Fluorescent signal	38	2	3	292
Matsuda K, 2011[35]	USA	Bloodstream infections	Pediatric patients	Blood	PCR-hybridization	Fluorescence pattern	122	11	1	94
Valle Jr DL, 2010[4]	Philippines	Bacteremia	Adult patients	Blood	PCR	400 bp	45	0	3	66
Chen LH, 2009[38]	China	Sepsis; meningitis	Children	Blood; CSF	FQ-PCR	C_T_ values ≤35 cycles	15	10	0	170
Handschur M, 2009[37]	Austria	Bloodstream infections	NR	Blood	PCR-sequencing	Sequence similarity>99.8%	7	0	2	13
Wellinghausen N, 2009[20]	Germany	Bacteremia	Adults and children	Blood	PCR	450 bp	47	41	7	247
Dutta S, 2009[14]	India	Sepsis	Neonates	Blood	PCR	380 bp	50	7	2	183
Ohlin A, 2008[18]	Sweden	Bacteremia	Neonates	Blood	real-time PCR	CP value with a range of 19–29.8	21	12	29	233
Wu YD, 2007[39]	China	Sepsis	Neonates	Blood	FQ-PCR	C_T_ values ≤35 cycles	20	23	0	787
Jordan JA, 2006[16]	USA	Sepsis	Neonates	Blood	PCR	380 bp	7	30	10	1186
Shang S, 2005[15]	China	Sepsis; meningitis	Neonates	Blood	PCR	371 bp	8	9	0	155
Tong MQ, 2004[40]	China	Sepsis	Neonates	Blood; CSF	PCR	371 bp	8	9	0	268
Shang S, 2001[41]	China	Sepsis	Neonates	Blood; CSF	PCR-hybridization	371 bp	26	0	0	30
Sleigh J, 2001[19]	New Zealand	Bacteremia	Adult patients	Blood	PCR	NR	15	48	13	121
Jordan JA, 2000[42]	USA	Bacteremia	Infants	Blood	PCR	380 bp	24	3	1	520
Draz NI, 2013[3]	Egypt	Sepsis	Neonates	Blood	PCR	1100 bp	20	15	8	7
Ohlin A, 2012[34]	Sweden	Sepsis	Neonates	Blood	real-time PCR	NR	44	31	12	281
Esparcia O, 2011[17]	Spain	Sepsis	Neonates	Blood	RT-qPCR	CT: between cycles 30 and 32	3	3	4	73
Fujimori M, 2010[5]	Japan	Sepsis	Neonates	Blood	RT-qPCR	NR	6	9	0	24
Yadav AK, 2005[44]	India	Sepsis	Neonates	Blood	PCR	861 bp	9	4	0	87
Makhoul IR, 2005[45]	Israel	late-onset sepsis	Neonates	Blood	PCR	10 CFU/ml of blood	9	0	4	202
Jordan JA, 2005[46]	USA	Sepsis	Neonates	Blood	real-time PCR	CT value >1.0	51	0	2	32
Laforgia N, 1997[43]	Italy	Sepsis	Neonates	Blood	PCR	861 bp	4	2	0	27
Reier-Nilsen T, 2009[36]	Norway	Sepsis	Infants	Blood	PCR	1500 bp, 1100 bp or 500 bp	4	6	2	36

TP, true positive; FP, false positive; FN, false negative; TN, true negative; CSF, cerebrospinal fluid; PCR, polymerase chain reaction; FQ-PCR, fluorescent quantitative polymerase chain reaction; RT-qPCR, reverse transcription-quantitative PCR; CP, Crossing Point; C_T_, cycle threshold; bp, base pairs; NR, no report.

**Fig 2:**
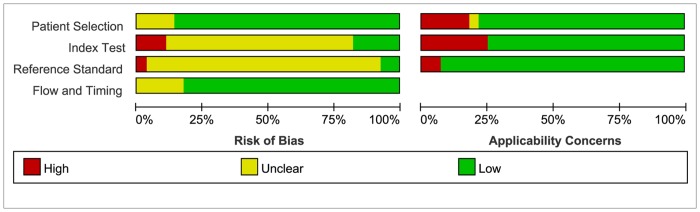
Risk of bias and applicability concerns graph. Review authors' judgments about each domain presented as percentages across included studies.

**Fig 3:**
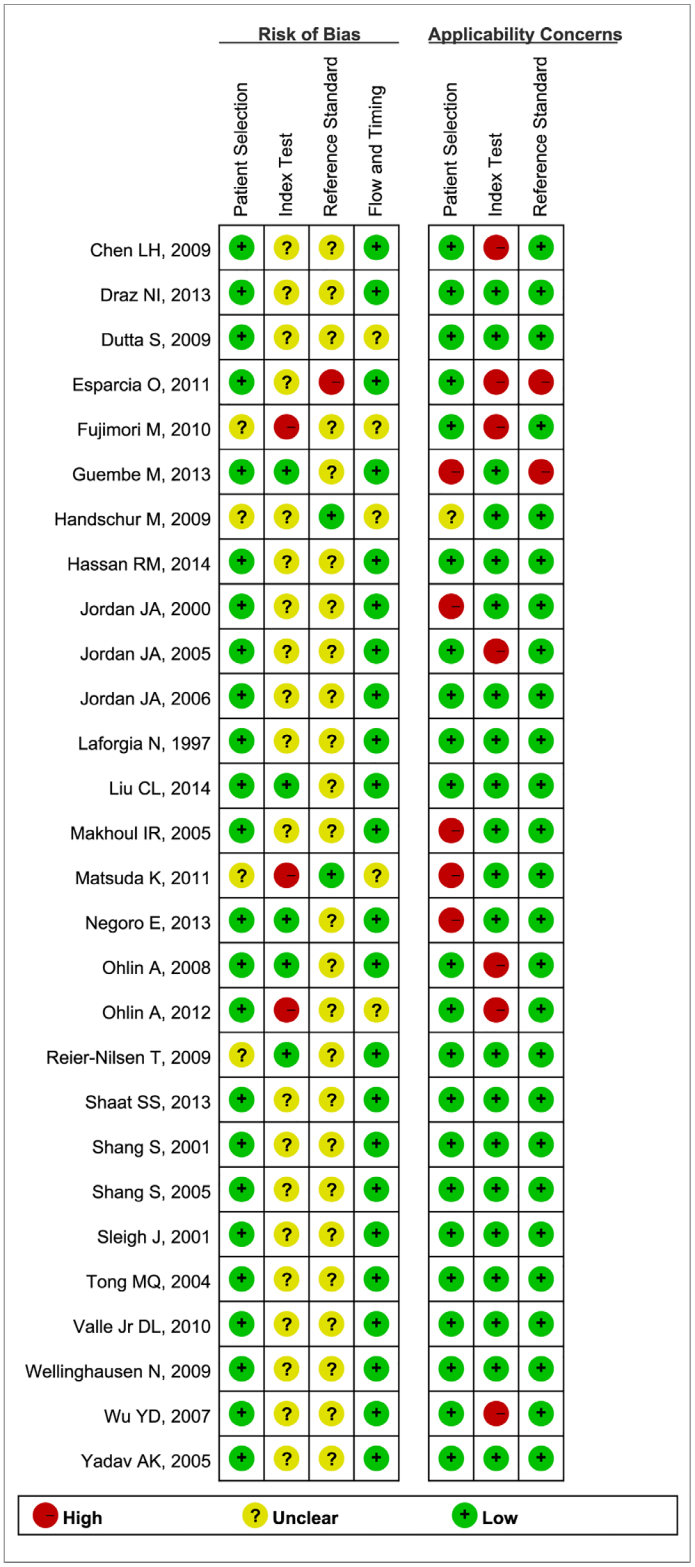
Risk of bias and applicability concerns summary. Review authors' judgments about each domain for each included study.

The detailed quality information of the included studies is shown in S3 Table. According to QUADAS-2 tool, 24 (85.7%) studies were at low risk of patient selection bias. A similar situation was observed in the flow and timing. As for index test and reference standard, the overwhelming majority (82.1% and 92.9%, respectively) studies were at high or unclear risk due to insufficient information to judge whether their test results were interpreted blind. Only 6 studies reported blinded interpretation of index test [1, 10, 12, 18, 19, 36], and 2 studies reported the blinded interpretation of reference standard [35, 37]. From an overall perspective, the qualities of the reported studies all turned out to be moderate to high concerns about applicability. Risk of bias and applicability concerns graph is presented in Fig 2. Risk of bias and applicability concerns summary is presented in Fig 3.

### Heterogeneity analysis and diagnostic accuracy

**Fig 4:**
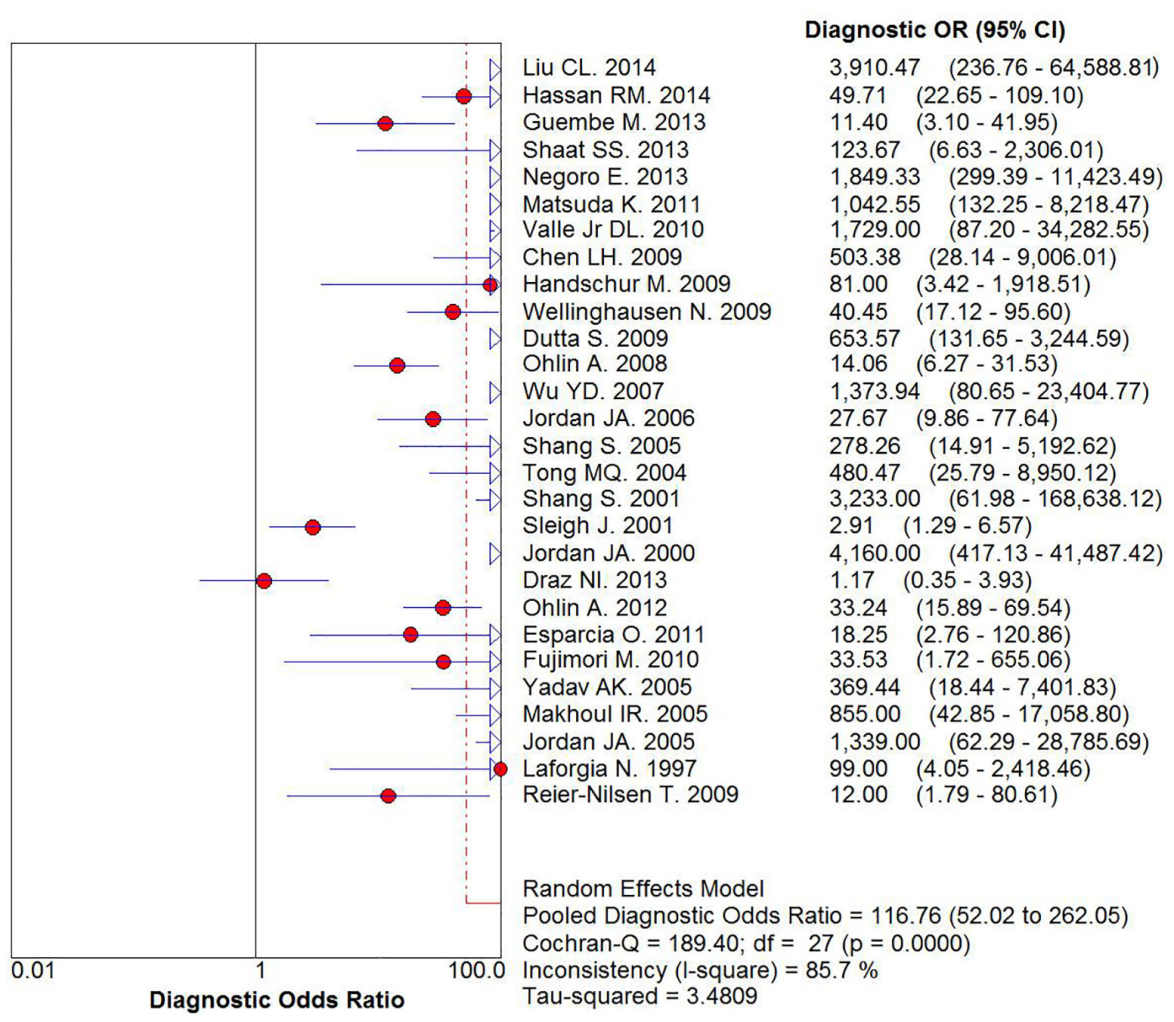
Diagnostic Odds Ratio with Cochran-Q value.

Spearman correlation coefficient of sensitivity and 1-specificity was found to be -0.177 with a ρ value of 0.367, indicating that there was no heterogeneity caused by the threshold effect. As was depicted in the Fig 4, statistically significant heterogeneity was observed when we pooled DOR of included studies. The result suggested that there should be other factors rather than threshold effect resulting in variations in accuracy estimates. We performed the univariable meta-regression analysis based on the publication year, sample size, disease type (sepsis or bacteremia), population characteristics (neonates or adult), and PCR test (qualitative or quantitative). Meta-regression analysis indicated that disease type and PCR test were significantly (p<0.05) associated with specificity and that population characteristics were significantly (p<0.05) related to the sensitivity. The detailed results of meta-regression analysis are presented in S4 Table and S1 Fig. Therefore, we decided that a random-effects model was used to eliminate some heterogeneity.

**Fig 5:**
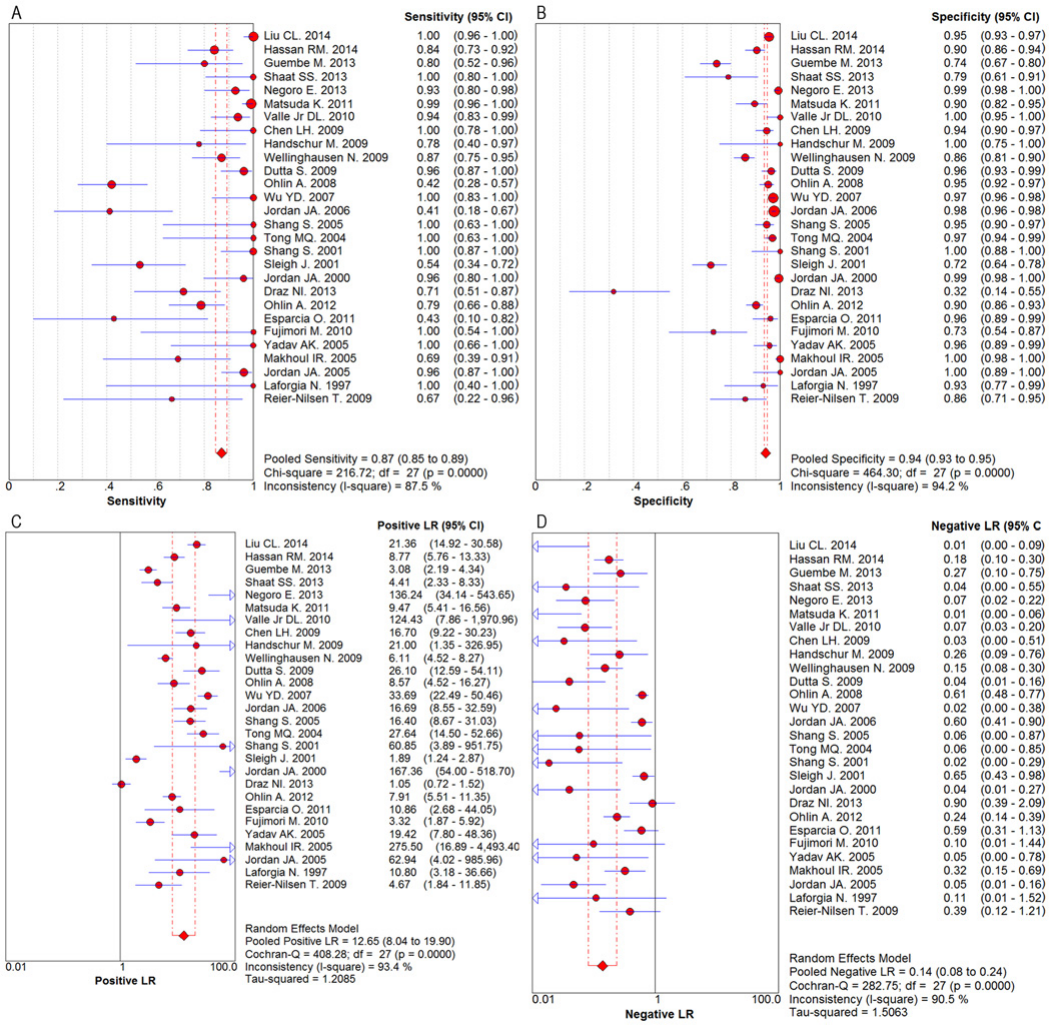
Forest plots for the diagnostic accuracy of 16S rRNA gene PCR. A. Sensitivity; B. Specificity; C. Positive likelihood ratio; D. Negative likelihood ratio.

**Fig 6:**
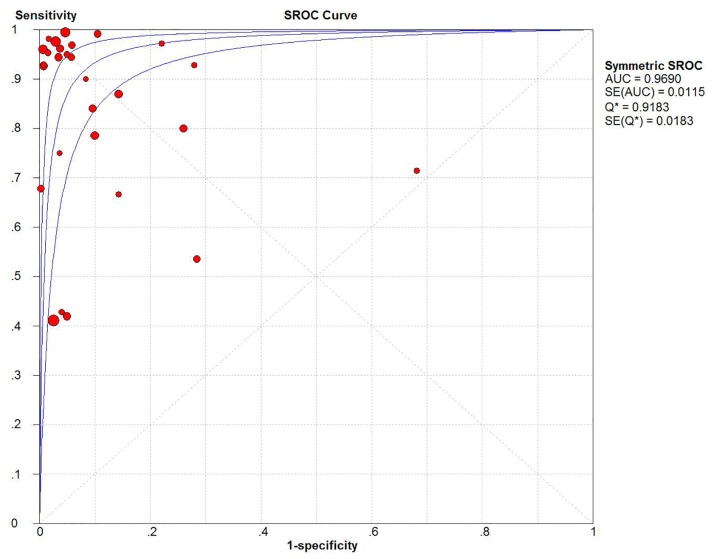
Summary receiver operating characteristic curve of 16S rRNA gene PCR diagnostic value in bloodstream infections.

After analysis using the random effect model, our meta-analysis showed that sensitivity and specificity were 0.87 (95% CI, 0.85–0.89) and 0.94 (95% CI, 0.93–0.95), respectively (Fig 5A and 5B). The results suggested that 16S rRNA gene PCR test had a higher specificity than sensitivity in the diagnosis of BSIs. The overall PLR was 12.65 (95% CI, 8.04–19.90) (Fig 5C), the overall NLR was 0.14 (95% CI, 0.08–0.24) (Fig 5D), and the pooled DOR was 116.76 (95% CI, 52.02–262.05) (Fig 4). The SROC curve for the included studies was shown in Fig 6. The pooled AUC and Q* of SROC curve were 0.9690 and 0.9183, respectively.

### Subgroup analysis

We also performed subgroup analyses according to population characteristics, disease type and test methods (Table 2). For the diagnostic accuracy of 16S rRNA gene PCR test for neonates, the sensitivity was 0.85 (95% CI, 0.81–0.88), the specificity was 0.96 (95% CI, 0.95–0.96), the PLR was 13.19 (95% CI, 7.08–24.57), and the NLR was 0.14 (95% CI, 0.06–0.29). Additionally, the AUC and Q* of SROC curve were 0.9714 and 0.9221. However, for adult patients, the sensitivity was 0.79 (95% CI, 0.69–0.87), the specificity was 0.77 (95% CI, 0.73–0.81), the PLR was 4.37 (95% CI, 1.17–16.34), and the NLR was 0.24 (95% CI, 0.05–1.14). Additionally, the AUC and Q* of SROC curve were 0.7668 and 0.7074, indicating a lower accuracy compared with neonates. As for the types of disease, the sensitivity of sepsis was significantly higher than bacteremia. Interestingly, compared with other amplification methods, PCR-hybridization showed a higher level of overall accuracy. After including 3 studies, the pooled sensitivity, specificity, PLR, and NLR (95% CI) increased to 0.98 (95% CI, 0.95–0.99), 0.97 (95% CI, 0.95–0.98), 37.98 (95% CI, 4.78–301.89), and 0.03 (95% CI, 0.01–0.13), respectively. Additionally, the AUC and Q* of SROC curve were 0.9958 and 0.9751.

**Table 2:** Summary of subgroup analysis of the included studies by different study characteristics.

Subgroups	No. of Studies	Sensitivity (95% CI)	Specificity (95% CI)	PLR (95% CI)	NLR (95% CI)	DOR (95% CI)	AUC	Q*
Overall	28	0.87 (0.85–0.89)	0.94 (0.93–0.95)	12.65 (8.04–19.90)	0.14 (0.08–0.24)	116.76 (52.02–262.05)	0.9690	0.9183
**Population characteristics**								
Neonates	17	0.85 (0.81–0.88)	0.96 (0.95–0.96)	13.19 (7.08–24.57)	0.14 (0.06–0.29)	121.17 (41.97–349.79)	0.9714	0.9221
Adult patients	3	0.79 (0.69–0.87)	0.77 (0.73–0.81)	4.37 (1.17–16.34)	0.24 (0.05–1.14)	25.86 (1.69–395.53)	0.7668	0.7074
**Disease type**								
Sepsis	15	0.90 (0.87–0.93)	0.95 (0.95–0.96)	11.36 (5.76–22.41)	0.12 (0.05–0.29)	108.89 (33.22–356.94)	0.9681	0.9168
Bacteremia	7	0.77 (0.71–0.82)	0.94 (0.93–0.95)	25.97 (7.54–89.42)	0.20 (0.08–0.47)	158.82 (24.81–1016.59)	0.9304	0.8656
**Test method**								
PCR	15	0.88 (0.84–0.91)	0.95 (0.94–0.95)	13.06 (6.42–26.58)	0.14 (0.06–0.32)	120.94 (31.82–459.64)	0.9653	0.9124
PCR-sequencing	3	0.83 (0.74–0.90)	0.83 (0.79–0.86)	6.17 (1.96–19.45)	0.20 (0.13–0.32)	30.61 (9.76–95.99)	0.8946	0.8255
PCR-hybridization	3	0.98 (0.95–0.99)	0.97 (0.95–0.98)	37.98 (4.78–301.89)	0.03 (0.01–0.13)	1568.82 (431.48–5703.98)	0.9958	0.9751
Real-time PCR	3	0.73 (0.65–0.80)	0.93 (0.90–0.95)	9.06 (4.77–17.21)	0.21 (0.06–0.76)	40.16 (9.68–166.52)	0.9458	0.8849

CI, confidence interval; PLR, positive likelihood ratio; NLR, negative likelihood ratio; DOR, diagnostic odds ratio; AUC area under the curve; Q*, Q point value.

### Publication bias

**Fig 7:**
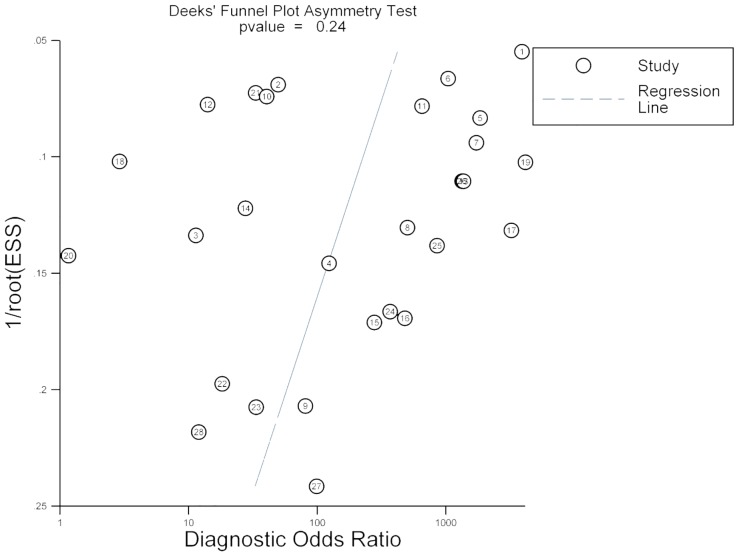
Funnel graph for the assessment of potential publication bias of the 28 included studies.

The Deeks’ test did not indicate any strong statistical evidence of publication bias, with ρ-value of 0.24 for the overall analysis. The shape of the funnel plot of the pooled DOR of 16S rRNA gene PCR in the diagnosis of BSIs also did not show any evidence of obvious asymmetry (Fig 7), indicating that there was no potential publication bias.

## Discussion

A previous meta-analysis had demonstrated that 16S rRNA gene PCR test had excellent sensitivity and specificity in the diagnosis of bacterial meningitis [47]. However, we did not know whether the test had a similar effect to BSIs. Studies focusing on the diagnostic value of 16S rRNA gene PCR test have been conducted in recent years. Thus, in this study we evaluated the accuracy of 16S rRNA gene PCR test in the diagnosis of BSIs.

Our results showed that the pooled sensitivity of the 16S rRNA gene PCR test was 0.87 and the pooled specificity was 0.94. This test may be a valid tool for confirming the diagnosis of BSIs, although not perfect. To illustrate the overall performance of 16S rRNA gene PCR test, we also counted the AUC and Q* of the SROC curve. A SROC curve is usually used to summarize overall test performance, while the AUC under the SROC curve is a measure of the overall performance of a diagnostic test to accurately differentiate those with and those without the condition of interest [30]. Q* is defined by the intercept of the SROC, which is closest to the ideal top-left corner of the SROC space and which corresponds to the highest value of sensitivity and specificity for the test [30, 48]. In present meta-analysis, the data showed that the AUC and Q* were 0.9690 and 0.9183, indicating very good ability to diagnose BSIs. The DOR reflects the relationship between the result of the diagnostic test and the disease, the value of which ranges from 0 to infinity—higher values indicating better discriminatory test performance [49]. Our meta-analysis showed that the pooled DOR was 116.76, suggesting a high level of overall accuracy. Compared with the DOR and SROC curve, the likelihood ratio (PLR and NLR) is considered to be more clinically meaningful for our measures of diagnostic accuracy [50]. The PLR represents the value by which the odds of the disease increase when a test is positive. Whereas NLR shows the value by which the odds of the disease decrease when a test is negative. The PLR value was 12.65 in the overall analysis, which suggested that patients with a positive PCR result had a about 13-fold chance of being diagnosed with BSIs rather than non-BSIs. On the other hand, the NLR was 0.14, which suggested that if a PCR result was negative, the probability rate of the individual having BSIs was 14% in theory.

Heterogeneity is a potential problem in interpreting the results of any meta-analysis. The threshold effect arises when differences in sensitivities and specificities occur due to different cut-offs or thresholds used in different studies to define a positive or negative test result [23]. We took the threshold effect as the first factor in our meta-analysis. We used the Spearman correlation coefficient to analyze the threshold effect. The result showed that Spearman value was found to be -0.177 (ρ = 0.367) using Meta-Disc analysis, suggesting that the heterogeneity was not caused by the threshold effect. However, the Cochran-Q value and I^2^ test showed that the heterogeneity among studies was too obvious to be ignored. To find the possible reasons for heterogeneity, we undertook a univariable meta-regression analysis based on the publication year, sample size, disease type (sepsis or bacteremia), population characteristics (neonates or adult), and PCR test (qualitative or quantitative). Unexpectedly, we found that disease type, population characteristics and PCR test were attributable to the sources of heterogeneity.

Thus, we performed the subgroup analyses according to population characteristics, disease type and test methods. Compared with the previous meta-analysis conducted by Pammi et al [51], we found that there was similar specificity (0.96, 95% CI: 0.94–0.97) and sensitivity (0.90, 95% CI: 0.78–0.95) to our subgroup analysis based on sepsis. It was noteworthy that PCR-hybridization test was more accurate in distinguishing patients with BSIs from non-BSIs people than other amplification methods, whereas the result should be interpreted with caution due to limited data and heterogeneity. But we did not conduct subgroup analyses based on the fluorescent quantitative PCR (FQ-PCR) and reverse transcription-quantitative PCR (RT-qPCR) owing to limited original data.

A previous study conducted by Loonen et al [52] suggested that deoxyribose nucleic acid (DNA) isolation methods could possibly affect BSIs diagnostics. In our meta-analysis, a reliable estimate of the amount of nucleic acid isolation was not provided in included studies, and extraction processes varied from boiling techniques [14, 15, 38–42]to differently commercial DNA extraction kits[1–4, 10, 17, 18, 20, 36, 37, 45], even RNA extraction kits [5]. Therefore, it was overwhelmingly difficult to compare the success of each of these methods.

Similar to other meta-analyses, several limitations should be acknowledged. Firstly, While the methodological quality of studies was assessed according to the QUADAS-2 tool, most studies were at unclear risk bias in index test and reference standard due to lacking of blinding results interpreted. Secondly, only published English and Chinese language studies were included in this meta-analysis, so the language bias might influence the results. Thirdly, the cut-off values varied widely, which made it difficult to determine the optimized cut-off value. Finally, Only 902 (12.2%) of 7,378 specimens had positive blood culture results, which could lead to broad variance about sensitivity.

In conclusion, our study is the first comprehensive meta-analysis to date that has assessed the accuracy of 16S rRNA gene PCR test in the diagnosis of BSIs. Despite the limitations mentioned above, the current evidence suggests that the 16S rRNA gene PCR test is a rapid, practical and valid tool for confirming the diagnosis of BSIs, especially sepsis. However, there is insufficient data to fully confirm diagnostic accuracy of PCR-hybridization test. Further meta-analysis involving more prospective studies with analysis of subgroups by amplification methods should be performed in the future.

## Supporting Information

S1 Fig Univariable meta-regression & Subgroup analysis. (TIF)

S1 PRISMA Checklist PRISMA checklist. (DOC)

S1 Table Search strategy. (DOC)

S2 Table Articles excluded along with the reasons for exclusion. (DOC)

S3 Table The detailed quality information of the included studies. (XLS)

S4 Table Meta-regression analyses of potential source of heterogeneity. (DOC)
