# Supplementary figures and images for: 16S Ribosomal Ribonucleic Acid Gene Polymerase Chain Reaction in the Diagnosis of Bloodstream Infections: A Systematic Review and Meta-Analysis
Source: PLoS One. 2015 May 21;10(5):e0127195. doi: 10.1371/journal.pone.0127195 (PMC4440735; doi:10.1371/journal.pone.0127195)

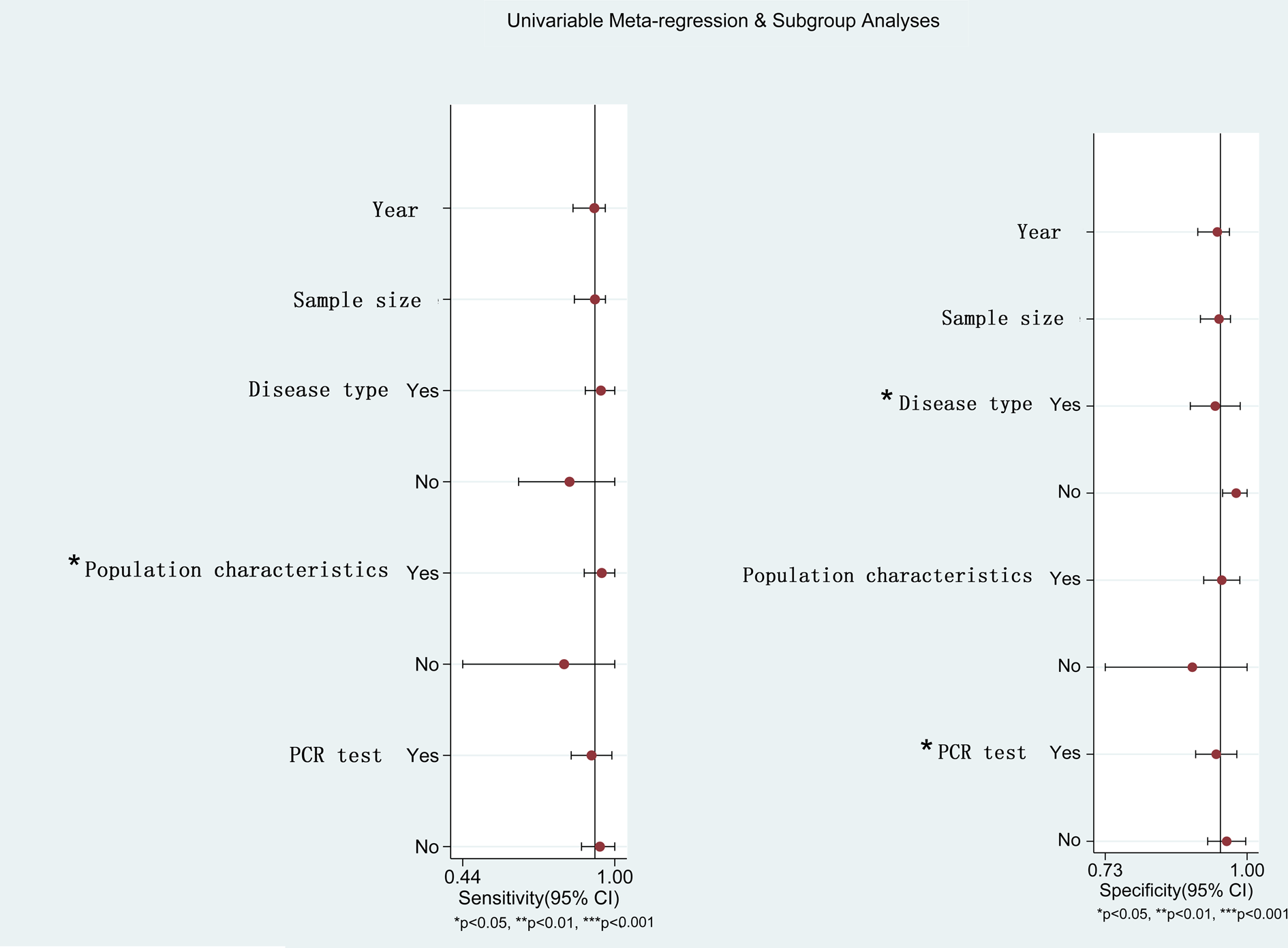

Supplement: S1 Fig — (TIF) [file pone.0127195.s001.tif]
