# Supplementary material for: 16S Ribosomal Ribonucleic Acid Gene Polymerase Chain Reaction in the Diagnosis of Bloodstream Infections: A Systematic Review and Meta-Analysis
Source: PLoS One. 2015 May 21;10(5):e0127195. doi: 10.1371/journal.pone.0127195 (PMC4440735; doi:10.1371/journal.pone.0127195)
Supplement: S1 Table — (DOC) [file pone.0127195.s003.doc]

**S1** **Table. Search strategy**

| **Database** | **Last Update** | **Search Strategy** | **results** |
| --- | --- | --- | --- |
| PubMed | March 14, 2015 | ((("rna, ribosomal, 16s"[MeSH Terms] OR (16S[All Fields] AND ("rna, ribosomal"[MeSH Terms] OR ("rna"[All Fields] AND "ribosomal"[All Fields]) OR "ribosomal rna"[All Fields] OR ("ribosomal"[All Fields] AND "ribonucleic"[All Fields] AND "acid"[All Fields]) OR "ribosomal ribonucleic acid"[All Fields]) AND ("genes"[MeSH Terms] OR "genes"[All Fields] OR "gene"[All Fields]))) OR (16S[All Fields] AND ("genes, rrna"[MeSH Terms] OR ("genes"[All Fields] AND "rrna"[All Fields]) OR "rrna genes"[All Fields] OR ("rrna"[All Fields] AND "gene"[All Fields]) OR "rrna gene"[All Fields]))) OR (16S[All Fields] AND ("dna, ribosomal"[MeSH Terms] OR ("dna"[All Fields] AND "ribosomal"[All Fields]) OR "ribosomal dna"[All Fields] OR "rdna"[All Fields]))) AND ((("sepsis"[MeSH Terms] OR (("blood circulation"[MeSH Terms] OR ("blood"[All Fields] AND "circulation"[All Fields]) OR "blood circulation"[All Fields] OR "bloodstream"[All Fields]) AND ("infection"[MeSH Terms] OR "infection"[All Fields] OR "infections"[All Fields]))) OR ("bacteraemia"[All Fields] OR "bacteremia"[MeSH Terms] OR "bacteremia"[All Fields])) OR ("septicaemia"[All Fields] OR "sepsis"[MeSH Terms] OR "sepsis"[All Fields] OR "septicemia"[All Fields])) | 807 |
| EMBASE | March 14, 2015 | #1 'rna, ribosomal, 16s'/exp OR 'rna, ribosomal, 16s' OR '16s ribosomal ribonucleic acid gene' OR '16s rrna gene' OR '16s rdna'/exp OR '16s rdna'  #2 'sepsis'/exp OR 'sepsis' OR 'bloodstream infections'/exp OR 'bloodstream infections' OR 'bacteremia'/exp OR 'bacteremia' OR 'septicemia'/exp OR 'septicemia'  #3 (#1 AND #2) | 894 |
| The Cochrane Library (Wiley) | Issue 2 of 12, February 2015 | #1 RNA, Ribosomal, 16S:ti,ab,kw or 16S ribosomal ribonucleic acid gene or 16S rRNA gene or 16S rDNA (Word variations have been searched)  #2 sepsis:ti,ab,kw or bloodstream infections or bacteremia or septicemia (Word variations have been searched)  #3 (#1 and #2) | 13 |
| ClinicalTrials.gov | March 15, 2015 | #1 "16S rRNA" AND "sepsis"  #2 "16S rRNA" AND "bloodstream infections"  #3 "16S rRNA" AND "bacteremia"  #4 "16S rRNA" AND "septicemia" | 0 |
| World Health Organization International Trials Registry Platform search portal | March 15, 2015 | #1 16S rRNA AND sepsis  #2 16S rRNA AND bloodstream infections  #3 16S rRNA AND bacteremia  #4 16S rRNA AND septicemia | 1 |
| Reference  lists | March 17, 2015 | Additional closely relevant articles were identified through hand search of bibliographies of retrieved studies and recent reviews. Hand search was limited by MeSH term (“RNA, Ribosomal, 16S” and “sepsis”) in the title. | 4 |
