# Supplementary material for: 16S Ribosomal Ribonucleic Acid Gene Polymerase Chain Reaction in the Diagnosis of Bloodstream Infections: A Systematic Review and Meta-Analysis
Source: PLoS One. 2015 May 21;10(5):e0127195. doi: 10.1371/journal.pone.0127195 (PMC4440735; doi:10.1371/journal.pone.0127195)
Supplement: S2 Table — (DOC) [file pone.0127195.s004.doc]

**S2 Table. Articles excluded along with the reasons for exclusion.**

| **First author** | **Year** | **Reason for exclusion** |
| --- | --- | --- |
| Deck MK (1) | 2014 | This study evaluated the accuracy of *Enterococcus Quick*FISH Method. |
| Zhang YC (2) | 2013 | This study explored the performance of loop-mediated isothermal amplification (LAMP) technology. |
| Rogina P (3) | 2014 | This study assessed the accuracy of CD64 index using 16S rRNA gene PCR as reference standard. |
| Park KS (4) | 2012 | This study did not assess the accuracy of 16S rRNA gene PCR test. |
| Muhl H (5) | 2010 | This study optimized PCR reagents with respect to the activity and DNA contamination. |
| Kim CM (6) | 2010 | This study evaluated oligonucleotide chip based on the 16S–23S rRNA gene. |
| Hansen WL (7) | 2010 | Species- or genus-specific probes were used 16S real-time PCR-based identification assay. Not evaluate diagnostic value of 16S rRNA gene PCR. |
| Zhao Y (8) | 2009 | This study evaluated the accuracy of RNA-dependent NASBA-MB detection system. |
| Xu J (9) | 2004 | The test was based on 16S rRNA gene and the 5.8, 18 and 28S rRNA combined with PCR. |
| Qian Q (10) | 2001 | Evaluation of BACTEC 9240 instrument using 16S rRNA gene PCR as reference standard. |
| Turenne CY (11) | 2000 | Identification using a fluorescence-based PCR-SSCP protocol. |
| MacGregor RR (12) | 1999 | Evaluate the sensitivity and specificity of Mycobacterium-specific PCR and HIV viral-load testing. |
| Wang HY (13) | 2014 | All specimens were directly from positive blood cultures, which did not have sufficient data to reconstruct 2 × 2 tables. |
| Motoshima M (14) | 2012 | All samples were from positive blood culture, which was not compared with the reference standard. |
| Schabereiter-Gurtner C (15) | 2008 | All blood cultures were negative, which did not have sufficient data to reconstruct 2 × 2 tables. |
| Schrenzel J (16) | 2007 | This study did not compare 16S rRNA gene PCR test with the reference standard. |
| Brozanski BS (17) | 2006 | Analysis included 419 infants with negative PCR and negative blood culture results. |
| Jordan JA (18) | 2005 | The purified bacterial isolates were included in this study, which did not have sufficient data to reconstruct 2 × 2 tables. |
| Woo PCY (19) | 2005 | All isolates were identified as *Clostridium perfringens*, which was not compared with the reference standard. |
| Christensen JE (20) | 2004 | *Lactobacillus* strains were isolated from positive clinical blood cultures. |
| Heininger A (21) | 2004 | 16S rRNA gene PCR assay was not compared with the reference standard. |
| Lee MS (22) | 2013 | Molecular diagnosis of periprosthetic joint infection. |
| Yassin AF (23) | 2012 | This study evaluated whether the developed real-time PCR is specific for *Tsukamurella*, which was not compared with the reference standard. |
| Jordan JA (24) | 2009 | All isolates were directly from positive blood culture bottles. |
| Shu XL (25) | 2008 | 16S rRNA gene real-time PCR assay was not compared with the reference standard. |
| Bahrani-Mougeot FK (26) | 2008 | Identification of oral bacteria. |
| Ammann RA (27) | 2007 | This study included pediatric cancer patients with fever and neutropenia. |
| Woo PCY (28) | 2001 | 16S ribosomal RNA gene PCR test was not compared with the reference standard. |
| Ahmet Z (29) | 1999 | Identification of *Group B b-hemolytic streptococci* in cerebrospinal fluid. |
| Ley BE (30) | 1998 | This study was not compared with the reference standard and did not have sufficient data to reconstruct 2 × 2 tables. |
| Kircher SM (31) | 1996 | This study assessed the accuracy for detecting group B Streptococci in obstetric patients. |
| Greisen K (32) | 1994 | The bacterial strains were used in this study, which was not compared with the reference standard. |
| Benitez-Paez A (33) | 2013 | The study group comprised five males and three females and contained no less than ten specimens. |
| Moriyama K (34) | 2008 | 16S ribosomal RNA genes (rDNA) were detected in blood samples from only two healthy individuals. |
| Stubljar D (35) | 2014 | Broad range 16S ribosomal RNA (rRNA) PCR was not compared with blood culture. |
| Jeng K (36) | 2012 | A case report. |
| Pammi M (37) | 2012 | A systematic review and meta-analysis. |
| Makhoul IR (38) | 2007 | Both blood cultures and 16S rRNA gene PCR test were negative in this study. |

**Reference List**

1. Deck MK, Anderson ES, Buckner RJ, Colasante G, Davis TE, Coull JM, et al. Rapid detection of Enterococcus spp. direct from blood culture bottles using Enterococcus QuickFISH Method: A multicenter investigation. Diagnostic microbiology and infectious disease. 2014;78(4):338-42.

2. Zhang YC. Pathogen diagnosis of children sepsis by LAMP technology. Asian Pacific journal of tropical medicine. 2013;6(3):242-5.

3. Rogina P, Skvarc M, Stubljar D, Kofol R, Kaasch A. Diagnostic utility of broad range bacterial 16S rRNA gene PCR with degradation of human and free bacterial DNA in bloodstream infection is more sensitive than an in-house developed PCR without degradation of human and free bacterial DNA. Mediators of inflammation. 2014;2014:108592.

4. Park KS, Ki CS, Kang CI, Kim YJ, Chung DR, Peck KR, et al. Evaluation of the GenBank, EzTaxon, and BIBI services for molecular identification of clinical blood culture isolates that were unidentifiable or misidentified by conventional methods. Journal of clinical microbiology. 2012;50(5):1792-5.

5. Muhl H, Kochem AJ, Disque C, Sakka SG. Activity and DNA contamination of commercial polymerase chain reaction reagents for the universal 16S rDNA real-time polymerase chain reaction detection of bacterial pathogens in blood. Diagnostic microbiology and infectious disease. 2010;66(1):41-9.

6. Kim CM, Song ES, Jang HJ, Kim HJ, Lee S, Shin JH, et al. Development and evaluation of oligonucleotide chip based on the 16S-23S rRNA gene spacer region for detection of pathogenic microorganisms associated with sepsis. Journal of clinical microbiology. 2010;48(5):1578-83.

7. Hansen WL, Beuving J, Bruggeman CA, Wolffs PF. Molecular probes for diagnosis of clinically relevant bacterial infections in blood cultures. Journal of clinical microbiology. 2010;48(12):4432-8.

8. Zhao Y, Park S, Kreiswirth BN, Ginocchio CC, Veyret R, Laayoun A, et al. Rapid real-time nucleic Acid sequence-based amplification-molecular beacon platform to detect fungal and bacterial bloodstream infections. Journal of clinical microbiology. 2009;47(7):2067-78.

9. Xu J, Moore JE, Millar BC, Alexander HD, McClurg R, Morris TCMC, et al. Improved laboratory diagnosis of bacterial and fungal infections in patients with hematological malignancies using PCR and ribosomal RNA sequence analysis. Leukemia and Lymphoma. 2004;45(8):1637-41.

10. Qian Q, Tang YW, Kolbert CP, Torgerson CA, Hughes JG, Vetter EA, et al. Direct identification of bacteria from positive blood cultures by amplification and sequencing of the 16S rRNA gene: evaluation of BACTEC 9240 instrument true-positive and false-positive results. Journal of clinical microbiology. 2001;39(10):3578-82.

11. Turenne CY, Witwicki E, Hoban DJ, Karlowsky JA, Kabani AM. Rapid identification of bacteria from positive blood cultures by fluorescence-based PCR-single-strand conformation polymorphism analysis of the 16S rRNA gene. Journal of clinical microbiology. 2000;38(2):513-20.

12. MacGregor RR, Dreyer K, Herman S, Hocknell PK, Nghiem L, Tevere VJ, et al. Use of PCR in detection of Mycobacterium avium complex (MAC) bacteremia: sensitivity of the assay and effect of treatment for MAC infection on concentrations of human immunodeficiency virus in plasma. Journal of clinical microbiology. 1999;37(1):90-4.

13. Wang HY, Kim S, Kim J, Park SD, Uh Y, Lee H. Multiplex real-time PCR assay for rapid detection of methicillin-resistant staphylococci directly from positive blood cultures. Journal of clinical microbiology. 2014;52(6):1911-20.

14. Motoshima M, Yanagihara K, Morinaga Y, Matsuda J, Hasegawa H, Kohno S, et al. Identification of bacteria directly from positive blood culture samples by DNA pyrosequencing of the 16S rRNA gene. Journal of medical microbiology. 2012;61(Pt 11):1556-62.

15. Schabereiter-Gurtner C, Nehr M, Apfalter P, Makristathis A, Rotter ML, Hirschl AM. Evaluation of a protocol for molecular broad-range diagnosis of culture-negative bacterial infections in clinical routine diagnosis. Journal of applied microbiology. 2008;104(4):1228-37.

16. Schrenzel J. Clinical relevance of new diagnostic methods for bloodstream infections. International journal of antimicrobial agents. 2007;30(SUPPL. 1):2-6.

17. Brozanski BS, Jones JG, Krohn MJ, Jordan JA. Use of polymerase chain reaction as a diagnostic tool for neonatal sepsis can result in a decrease in use of antibiotics and total neonatal intensive care unit length of stay. Journal of perinatology : official journal of the California Perinatal Association. 2006;26(11):688-92.

18. Jordan JA, Butchko AR, Durso MB. Use of pyrosequencing of 16S rRNA fragments to differentiate between bacteria responsible for neonatal sepsis. The Journal of molecular diagnostics : JMD. 2005;7(1):105-10.

19. Woo PCY, Lau SKP, Chan KM, Fung AMY, Tang BSF, Yuen KY. Clostridium bacteraemia characterised by 16S ribosomal RNA gene sequencing. Journal of Clinical Pathology. 2005;58(3):301-7.

20. Christensen JE, Reynolds CE, Shukla SK, Reed KD. Rapid molecular diagnosis of lactobacillus bacteremia by terminal restriction fragment length polymorphism analysis of the 16S rRNA gene. Clinical medicine & research. 2004;2(1):37-45.

21. Heininger A, Binder M, Ellinger A, Pfisterer J, Botzenhart K, Unertl K, et al. Effect of comprehensive validation of the template isolation procedure on the reliability of bacteraemia detection by a 16S rRNA gene PCR. Clinical Microbiology and Infection. 2004;10(5):452-8.

22. Lee MS, Chang WH, Chen SC, Hsieh PH, Shih HN, Ueng SWN, et al. Molecular diagnosis of periprosthetic joint infection by quantitative RT-PCR of bacterial 16S ribosomal RNA. The Scientific World Journal. 2013;2013.

23. Yassin AF, Muller J. Development of real-time polymerase chain reaction assay for specific detection of Tsukamurella by targeting the 16S rRNA gene. Diagnostic microbiology and infectious disease. 2012;72(3):219-25.

24. Jordan JA, Jones-Laughner J, Durso MB. Utility of pyrosequencing in identifying bacteria directly from positive blood culture bottles. Journal of clinical microbiology. 2009;47(2):368-72.

25. Shu XL, Wu YD, Shang SQ. Establishment of quantifying and typing analysis of 16S rRNA gene by real-time PCR. Zhongguo dang dai er ke za zhi = Chinese journal of contemporary pediatrics. 2008;10(6):732-6.

26. Bahrani-Mougeot FK, Paster BJ, Coleman S, Ashar J, Knost S, Sautter RL, et al. Identification of oral bacteria in blood cultures by conventional versus molecular methods. Oral surgery, oral medicine, oral pathology, oral radiology, and endodontics. 2008;105(6):720-4.

27. Ammann RA, Zucol F, Aebi C, Niggli FK, Kuhne T, Nadal D. Real-time broad-range PCR versus blood culture. A prospective pilot study in pediatric cancer patients with fever and neutropenia. Supportive Care in Cancer. 2007;15(6):637-41.

28. Woo PCY, Fung AMY, Lau SKP, Wong SSY, Yuen KY. Group G beta-hemolytic Streptococcal bacteremia characterized by 16S ribosomal RNA gene sequencing. Journal of clinical microbiology. 2001;39(9):3147-55.

29. Ahmet Z, Stanier P, Harvey D, Holt D. New PCR primers for the sensitive detection and specific identification of group B beta-hemolytic streptococci in cerebrospinal fluid. Molecular and cellular probes. 1999;13(5):349-57.

30. Ley BE, Linton CJ, Bennett DM, Jalal H, Foot AB, Millar MR. Detection of bacteraemia in patients with fever and neutropenia using 16S rRNA gene amplification by polymerase chain reaction. European journal of clinical microbiology & infectious diseases : official publication of the European Society of Clinical Microbiology. 1998;17(4):247-53.

31. Kircher SM, Meyer MP, Jordan JA. Comparison of a modified DNA hybridization assay with standard culture enrichment for detecting group B streptococci in obstetric patients. Journal of clinical microbiology. 1996;34(2):342-4.

32. Greisen K, Loeffelholz M, Purohit A, Leong D. PCR primers and probes for the 16S rRNA gene of most species of pathogenic bacteria, including bacteria found in cerebrospinal fluid. Journal of clinical microbiology. 1994;32(2):335-51.

33. Benitez-Paez A, Alvarez M, Belda-Ferre P, Rubido S, Mira A, Tomas I. Detection of transient bacteraemia following dental extractions by 16S rDNA pyrosequencing: a pilot study. PloS one. 2013;8(3):e57782.

34. Moriyama K, Ando C, Tashiro K, Kuhara S, Okamura S, Nakano S, et al. Polymerase chain reaction detection of bacterial 16S rRNA gene in human blood. Microbiology and immunology. 2008;52(7):375-82.

35. Stubljar D, Skvarc M. Expression of CD64 on neutrophils can be used to predict the severity of bloodstream infection before broad range 16S rRNA PCR. Folia microbiologica. 2014.

36. Jeng K, Yang S, Won H, Gaydos CA, Hsieh YH, Kecojevic A, et al. Application of a 16S rRNA PCR-high-resolution melt analysis assay for rapid detection of Salmonella Bacteremia. Journal of clinical microbiology. 2012;50(3):1122-4.

37. Pammi M, Flores A, Leeflang M, Versalovic J. Molecular assays in the diagnosis of neonatal sepsis: a systematic review and meta-analysis. Pediatrics. 2011;128(4):e973-85.

38. Makhoul IR, Sprecher H, Smolkin T, Sawaid R, Ben-David S, Sujov P, et al. Approach to term neonates born after maternal intrapartum fever and unknown maternal group B Streptococcus status: value of serum C-reactive protein and 16S rRNA gene PCR amplification. The Pediatric infectious disease journal. 2007;26(11):1064-6.
