# Supplementary material for: 16S Ribosomal Ribonucleic Acid Gene Polymerase Chain Reaction in the Diagnosis of Bloodstream Infections: A Systematic Review and Meta-Analysis
Source: PLoS One. 2015 May 21;10(5):e0127195. doi: 10.1371/journal.pone.0127195 (PMC4440735; doi:10.1371/journal.pone.0127195)
Supplement: S4 Table — (DOC) [file pone.0127195.s006.doc]

S4 Table. Meta-regression analyses of potential source of heterogeneity.

| **Parameter** | **Category** | **NO. of studies** | **Sensitivity** | **p1** | **Specificity** | **p2** |
| --- | --- | --- | --- | --- | --- | --- |
| Year |  | 28 | 0.93 [0.85 - 0.97] | 0.95 | 0.94 [0.91 - 0.97] | 0.74 |
| Sample size |  | 28 | 0.93 [0.85 - 0.97] | 1.00 | 0.95 [0.91 - 0.97] | 1.00 |
| Disease type | Yes | 15 | 0.95 [0.89 - 1.00] | 0.24 | 0.94 [0.89 - 0.99] | **0.03** |
|  | No | 7 | 0.83 [0.65 - 1.00] |  | 0.98 [0.95 - 1.00] |  |
| Population characteristics | Yes | 17 | 0.95 [0.89 - 1.00] | **0.05** | 0.95 [0.92 - 0.99] | 0.43 |
|  | No | 3 | 0.81 [0.44 - 1.00] |  | 0.90 [0.73 - 1.00] |  |
| PCR test | Yes | 18 | 0.91 [0.84 - 0.99] | 0.28 | 0.94 [0.90 - 0.98] | **0.01** |
|  | No | 10 | 0.95 [0.88 - 1.00] |  | 0.94 [0.90 - 0.98] |  |
